# Concerns and Considerations Regarding the Study on Epicardial Fat Thickness as a Marker for Coronary Artery Disease in Diabetic Patients

**DOI:** 10.1002/clc.70205

**Published:** 2025-12-06

**Authors:** Hakan Tibilli, Sezer Markirt

**Affiliations:** ^1^ Department of Cardiology Başkent University Adana Hospital Adana Türkiye; ^2^ Department of Cardiology Adıyaman University Training and Research Hospital Adıyaman Türkiye

Dear Editor,

We read with great interest the article titled “Epicardial Fat Thickness as a Marker of Coronary Artery Disease in Diabetics: A Single Center Study” [1]. The authors highlight significant findings regarding epicardial fat thickness (EFT) as an effective marker of coronary artery disease (CAD) severity in diabetic patients. However, several methodological and analytical aspects warrant further clarification and discussion.

Firstly, the exclusion criteria used in the study notably omit consideration of chronic kidney disease (CKD), frequently comorbid with diabetes mellitus and potentially impactful on both CAD prevalence and EFT [2]. Additionally, the retrospective design of this study raises critical questions regarding the practicality and reliability of assessing CAD presence and severity in a substantial patient cohort (*n* = 2340). The authors describe employing invasive and noninvasive tests retrospectively based on clinical indications, but the specifics of these indications and the process for systematically retrieving and validating past clinical data remain unclear. This approach might introduce substantial bias related to clinician‐dependent test selection.

Moreover, despite collecting detailed lipid profiles, the study did not address statin therapy usage, an essential consideration given its widespread use and known influence on CAD progression and potentially EFT itself. Future investigations would benefit from clarifying the role of statins or other pharmacotherapies that may influence EFT and CAD outcomes.

In summary, while this study provides insightful findings that underscore EFT's potential as a noninvasive cardiovascular marker, addressing these methodological and analytical concerns would considerably enhance the study's robustness and clinical applicability.

Sincerely,

## Data Availability

Data sharing is not applicable for this article.
